# Free Sugars Consumption in Canada

**DOI:** 10.3390/nu13051471

**Published:** 2021-04-27

**Authors:** Huma Rana, Marie-Claude Mallet, Alejandro Gonzalez, Marie-France Verreault, Sylvie St-Pierre

**Affiliations:** 1 Office of Nutrition Policy and Promotion, Health Canada, 100 Eglantine, Ottawa, ON K1A 0K9, Canada; alejandro.gonzalez-delgado@canada.ca (A.G.); sylvie.st-pierre2@canada.ca (S.S.-P.); 2 Food Directorate, Health Canada, 251 Sir Frederick Banting, Ottawa, ON K1Y 0M1, Canada; marie-claude.mallet@canada.ca (M.-C.M.); marie-france.verreault@canada.ca (M.-F.V.)

**Keywords:** free sugars, Canada, added sugars, total sugars, dietary recommendation

## Abstract

Free sugars (FS) are associated with a higher risk of dental decay in children and an increased risk of weight gain, overweight and obesity and type 2 diabetes. For this reason, Canada’s Food Guide recommends limiting foods and beverages that contribute to excess free sugars consumption. Estimating FS intakes is needed to inform policies and interventions aimed at reducing Canadians’ consumption of FS. The objective of this study was to estimate FS intake of Canadians using a new method that estimated the free sugars content of foods in the Canadian Nutrient File, the database used in national nutrition surveys. We define FS as sugars present in food products in which the structure has been broken down. We found that 12% of total energy (about 56 g) comes from FS in the diet of Canadians 1 year of age and older (≥1 year). The top four sources were: (1) sugars, syrups, preserves, confectionary, desserts; (2) soft drinks; (3) baked products and (4) juice (without added sugars), and accounted for 60% of total free sugars intake. The results show that efforts need to be sustained to help Canadians, particularly children and adolescents, to reduce their FS intake.

## 1. Introduction

**Figure 1:**
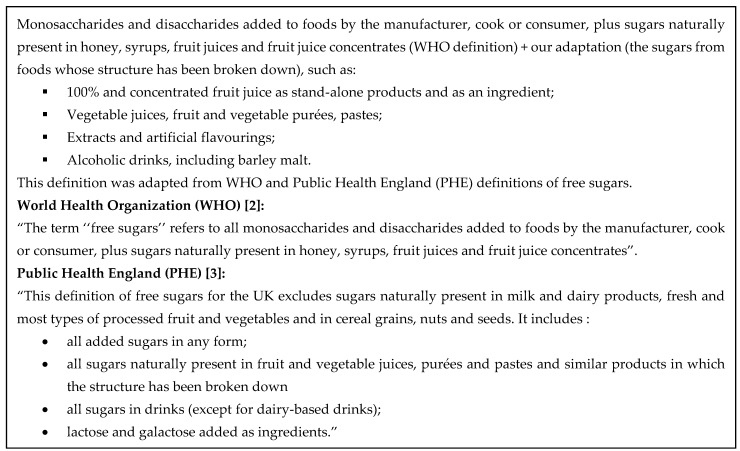
Free sugars definition used in this study.

Sugars are a type of carbohydrate found naturally in fruit, vegetables and milk. Sugars are added to foods to impart or increase sweetness, for preservation, or confer several functional characteristics such as viscosity, texture and color [1]. Free sugars (FS) are naturally present in honey, syrups, fruit juices and fruit juice concentrates [2]. FS do not include the naturally occurring sources of sugars found in intact or cut fruit and vegetables and (unsweetened) milk [2]. Monosaccharides and disaccharides added to foods and drinks by the manufacturer, cook or consumer are also considered free sugars (Figure 1) [3]. In order to captures all sources of free sugars in the diet of Canadians, we used an adapted FS definition for this study; see Figure 1 for details. Our definition considers foods in which the structure has been broken down as foods that contain free sugars. Using this methodology will enable the estimation of FS consumption of Canadians in a consistent manner for the purpose of informing healthy eating policies.

Evidence on the links between diet and health show that the intake of FS—particularly in the form of sugary drinks (including 100% fruit juice)—has been associated with a higher risk of dental decay in children [4].

Further, foods containing added sugars have been associated with an increased risk of weight gain, overweight and obesity and type 2 diabetes [4,5,6,7,8]. 

FS are highly prevalent in the Canadian food supply. However, it is difficult for consumers to identify their food sources, as they are not listed on food labels. This is because there is no analytical method to distinguish FS from other sugars that also occur in foods. A study by Bernstein et al. showed that, on average, FS represents 62% of the total sugars amount in prepackaged foods in the Canadian food supply [9].

Guidance on the consumption of sugar differs around the world. In 2015, the World Health Organization (WHO) published its ‘Sugar intake for adults and children’ Guideline recommending a FS intake of less than 10% of total energy intake to reduce the risk of noncommunicable diseases [2]. The United States’ Dietary Guidelines Advisory Committee recommends “Americans should keep their intake of added sugars to less than 10% of their total daily calories as part of a healthy diet” [7]. This recommendation for added sugars allows a larger amount of free sugar consumption as part of the daily intake, because it excludes 100% fruit juice. The United Kingdom’s Scientific Advisory Committee on Nutrition recommends that “Free sugars should account for no more than 5% daily dietary energy intake” [4]. In line with the guidance based on a strong recommendation by the WHO, Canada’s Dietary Guidelines support a FS intake of <10%E [10]. 

The main objective of this study was to estimate the FS intake in various Canadian age–sex groups using data from 2015 Canadian Community Health Survey (CCHS)-Nutrition. A methodology that could be applied to estimate the free sugar contents of foods in the 2015 Canadian Nutrient File (CNF) was developed for this purpose. The 2015 CNF database is a standard reference food composition database reporting the amount of nutrients in composite foods commonly consumed in Canada [11]. Composite foods report a national sales weighted average of nutritional information derived by combining multiple products (e.g., multiple skim milk brands) into a single composite profile (e.g., skim milk) that represents skim milk available in the Canadian food supply.

The secondary objectives of this study were:

1. To describe and compare FS intakes among various sociodemographic groups and by the body mass index (BMI).
2. To identify the top food sources of FS for various age–sex groups.

## 2. Materials and Methods

Dietary intake data from the 2015 CCHS-Nutrition were used along with the food composition data from the 2015 CNF database. 

The 2015 CCHS-Nutrition was a cross-sectional survey conducted by Statistics Canada, with data collected from January to December 2015 [12]. The target population is Canadian household residents aged 1 year and older living in one of Canada’s 10 provinces. Their sociodemographic, health-related information was collected through a questionnaire. Participants were also asked to report all foods and drinks consumed in the 24 h prior to the interview. The Automated Multiple Pass Method (AMPM) was used to collect the dietary intake data. The AMPM is a questionnaire that guides the interviewer to maximize the respondents’ recall of all foods consumed in the previous 24 h [12] A subsample of approximately 30% of respondents was randomly selected for a second interview between 3 to 10 days after the first one. More information on the 2015 CCHS-Nutrition is available elsewhere [12].

### 2.1. Methodology for Use with CNF

**Figure 2:**
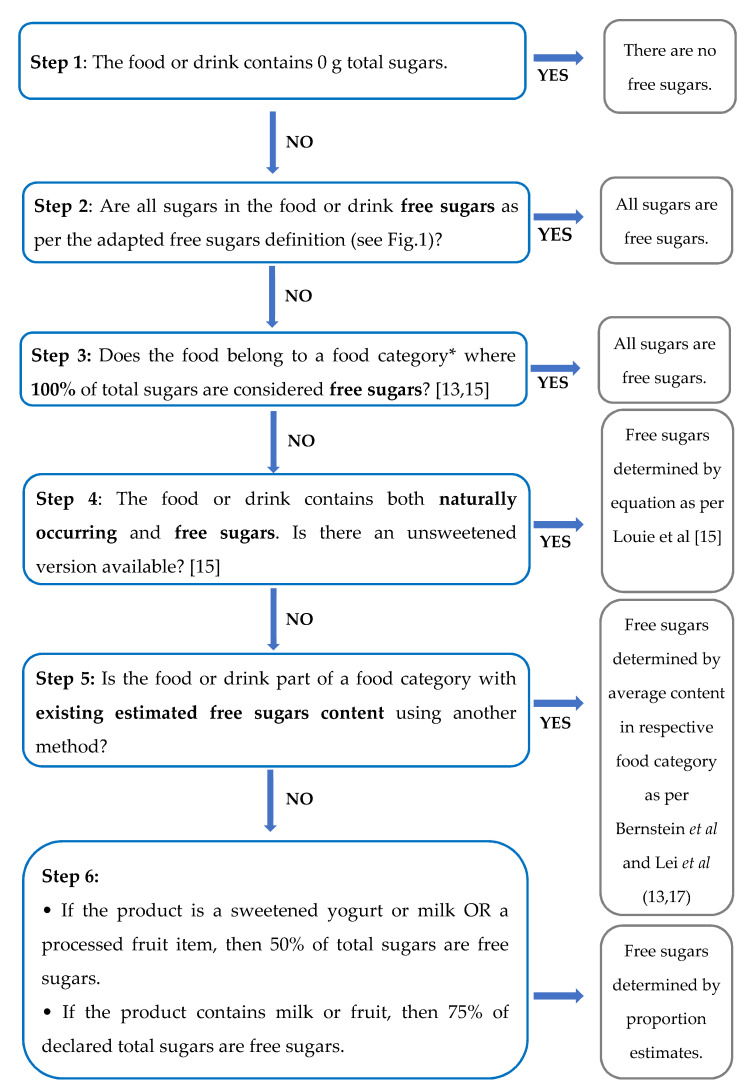
Flowchart assessing free sugars content in Foods without an ingredient list.

We reviewed the existing methodologies and explored options to assess the FS in foods [13,14,15,16,17]. Most of these methods require ingredient lists of foods to estimate the FS. Consequently, they could not be used directly on foods in the CNF, because this database is comprised of composite foods and, therefore, does not contain this information. A 6-step methodology was thus developed to estimate the FS content of the 2015 CNF food composition database in the absence of ingredient lists (see Figure 2). 

Pre-step: Two registered dietitians assessed the presence of naturally occurring sugars, added sugars, free sugars and sugar substitutes of all foods in the CNF database prior to applying the 6-step process.

* Categories of foods (used in Step 3) where 100% of total sugars are considered free sugars [9,13]:

- Cake mixes, Coffee Cake, Sponge Cakes
- All types of Cookies
- Energy Drinks, Fruit Drinks, Sweetened Flavored coffee, Soft Drinks, Sports drinks, Alcohol Mixes
- High-fiber Compact, Puffed, Shredded cereals
- Condensed Milk
- Desserts: Gelatin
- Ice Pops, Juice Bars, Cups
- Sweetened toppings and fillings (e.g., cake frosting)
- Fruit Garnish (e.g., maraschino cherries)
- Sweetened processed meats (e.g., maple-glazed bacon), Deli Meats and Sausages
- Seasoned/sweetened tofu and tempeh
- Baked Beans
- Barbecue, Steak Sauce, Mustard, Hot Sauces, Marinades, Sweet Sauce
- Sweet popcorn, Coated/filled Pretzels
- Dry Mix, Liquid concentrated bouillons and broths
- Confectionaries, Sugar, Sweet Condiments

Consensus was reached for all decisions when discrepancies occurred. The FS estimates from the 6-step methodology were then applied to the food intake data from the 2015 CCHS-Nutrition. The contribution of FS and %E from FS for the Canadian population was estimated. Meal replacement drinks and powders were excluded from these estimates, as these products fall under therapeutic dietary regimens. The Supplementary Materials include a separate file with our FS estimates of CNF foods.

### 2.2. Subjects

In the 2015 CCHS-Nutrition, 19,218 Canadians aged ≥ 1 year reported intakes of food. Participants with missing values of FS for at least one food (*n* = 1), those who were pregnant (*n* = 114), or breastfeeding (*n* = 185) were excluded from all the analyses. Height and weight were only measured for a subsample of participants aged ≥ 2 years. After previous exclusions, 5791 participants were identified with missing BMI due to missing height, weight or both measures and were excluded from BMI-related analyses.

### 2.3. Statistical Analysis

Statistical analyses were performed using the shared file through Statistics Canada using SAS EG version 7.1 (SAS Institute Inc, Cary, USA) [18,19]. Means and percentages were estimated using the first 24-h recall only. The PROC SURVEYREG procedure was used to estimate means of FS and %E from FS, overall, by categories within sociodemographic characteristics and by the BMI. The Wald’s F test was used to perform overall comparisons, while *t*-tests were used for comparisons between categories. Bonferroni corrections were applied to adjust for multiple comparisons. To meet the normality assumption of the models, a Box-Cox transformation of the outcome variable was implemented (i.e., FS, energy from FS). In order to estimate the percent of the population below 10%E from FS, respectively, the NCI method (amount-only model) was used [20]. The univariate approach was used in this study for consistency purpose with the rest of the analyses.

To identify the top sources of FS, the PROC SURVEYMEANS procedure, along with the RATIO statement, was used. To account for the complex sampling design and nonresponse, all estimates were weighted to be representative of the Canadian population.

In analyses involving BMI, special survey and bootstrap weights (also available through the survey) were used to account for missing height or weight responses. For the variance estimations, the Balanced Repeated Replication method was used in all analyses.

## 3. Results

### 3.1. Free Sugars Intake Overall by Sociodemographic Groups and BMI

Table 1 shows estimates of FS intakes as %E from FS and in grams by sociodemographic characteristics and by BMI. In the overall population (i.e., ≥1 year), the mean intake of FS was 12%E (or 56 g). Men reported significantly higher absolute average intake of FS (i.e., in grams) than women, 64 g to 48 g, respectively. However, their relative average intakes (i.e., %E from FS) show similar values, with 12%E in both groups (*p*-value = 0.53). Our data showed that, overall, approximately 55% of the total sugars intake came from FS. See Appendix A Table A1 for results of *t*-tests that show between group differences.

The analysis by age groups shows that the highest average intake of FS was by adolescents. The 9–13 years and 14–18 years reported the highest relative average intakes (about 15%E from FS), followed by the 1–8 years (14%E) and adults ≥19 years (11%E). Similarly, when looking at the absolute intake, the highest consumers were adolescents aged 14–18 years (80 g), followed by children aged 9–13 years (75 g). Children 1–8 years reported FS intake of 54 g and adults ≥19 years reported 53 g.

In the overall population, 40% of Canadians ≥1 year reported <10%E from FS in line with the WHO recommendation (results not shown). In children 1–8 y, this fell to 22%. Children and adolescents aged 9–13 years and 14–18 years were the least likely to meet the recommendation with 14% and 18%, respectively, consuming <10% from the FS. However, these results should be interpreted with caution due to the large variability in the estimates. 45% of adults ≥19 years meet the <10%E from FS recommendation.

The analysis by BMI groups did not suggest that there were any differences in the relative intake (*p*-value = 0.023) or the absolute average intake (*p*-value = 0.17) of FS. The %E from FS for people living in both urban and rural areas was approximately 12%. Individuals living in urban centers reported higher average intakes of FS (62 g) than those living in rural areas (55 g). Although the *p*-value for intake in grams suggests that the differences between groups is significant (*p*-value = 0.02), the difference appears to be small (<1%). Individuals with a university degree or higher reported a lower mean intake in both absolute and relative intakes of FS (53 g, 11%E) compared to those without a university degree or diploma (58 g, 12%E) with *p*-values of 0.005 and <0.001, respectively. The analysis of the FS intake in grams by income groups showed no significant difference (between 54 g and 57 g in all groups), with a *p*-value = 0.58. When considering the %E from FS, there was a difference from the highest to the lowest income quartile (from 12.3% to 10.7%) with a *p*-value ≤ 0.001.

Overall, in all groups the mean estimated %E from FS exceeds the WHO recommendation of <10%E from FS.

### 3.2. Top Sources of Free Sugars by Age-Sex Groups in 2015

**Figure 3:**
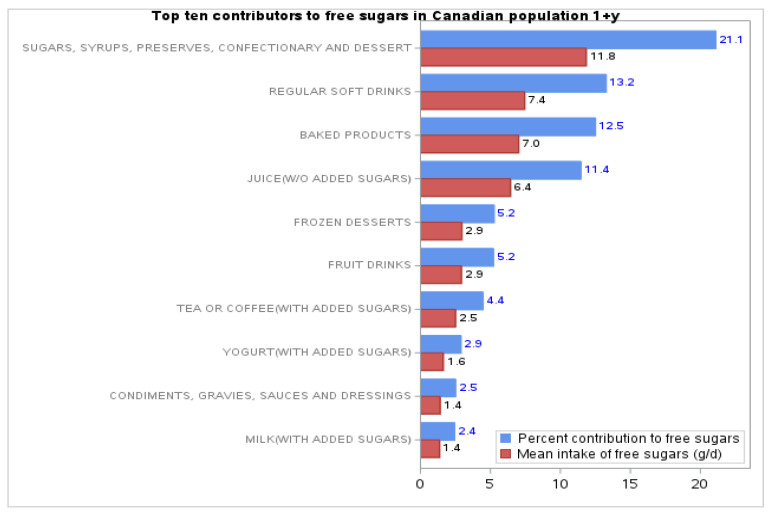
Top 10 contributors of free sugar intakes in Canadians 1 year or older.

The top 10 sources of FS in the diet of Canadians accounted for 81% of the total intake of FS in the overall population. The top four sources were: (1) sugars, syrups, preserves, confectionary, desserts; (2) soft drinks; (3) baked products and (4) juice (without added sugars). These top four sources accounted for 60% of the total free sugars intake. Together, sugary drinks (including soft drinks, juice without added sugars, beverages, e.g., tea or coffee with added sugars, energy drinks and alcohol) accounted for 40% of the FS intake from all sources (See Figure 3).

Comparisons by sex within the same age groups show very similar results for the top sources of FS (results not shown). In all age groups, as well as in the overall population, there was little difference in the relative amount of FS consumed from the top 10 sources of FS between men and women. The main difference was that men ≥ 19 y reported 6% more FS from sugary drinks than women (17% and 11%, respectively). In addition, the top four sources are almost identical in all groups, although in a different order. Women consumed more FS from sources such as “sugars, syrups, preserves, confectionary, desserts’ and “baked products” than men.

## 4. Discussion

To our knowledge, this study is the first to estimate the FS consumption of Canadians in all age–sex groups using data from 2015 CCHS-Nutrition combined with the Canadian food supply data.

The results from this study illustrate the FS intake in the Canadian population by age–sex, BMI and sociodemographic characteristics. Overall, our analyses of CCHS-Nutrition (2015) show a mean intake of free sugars of 12%E in the Canadian population (≥1 y), which is higher than WHO recommendation. Usual intake distributions revealed that only 40% of Canadians consume <10%E. In order to verify the impact of our adapted free sugars definition, we also compared the mean intakes of FS when applying the WHO definition vs. our adapted WHO definition. As expected, due to our comprehensive definition that included all sources of FS, the absolute intake in grams was higher. However, the relative intakes (%E) remained unchanged for the most part (See Appendix A Table A2 for a comparative data table).

Further analyses indicate slight differences in the FS consumption by age and sex. Men appear to consume more FS in grams; however, their %E from FS is similar to women. This can be explained by the fact that men have higher energy intakes. One of the key findings of our analyses, by age groups, is that adolescents (9–13 years and 14–18 years) are the highest consumers of FS, with a mean of about 15%E from FS. Less than 14% of 9–13 years and 18% 14–18 years consumed <10%E from FS. The proportion of children (1–8 years) meeting the <10%E recommendation is also low (22%). One of the reasons for this high intake of FS is the advertising of highly processed foods and beverages, which tend to be high in FS, to this age group, which are an important driver of childhood obesity [21].

Other Canadian-based studies showed similar results [14,15,22,23] A study by Bergeron et al. 2019 in French-speaking adults ≥18 years in the province of Québec reported an average of 12%E consumption from FS, which is close to our results (11%E in adults) [14] Additionally, a study by Wang et al. published in 2020, using 2015 CCHS-Nutrition data as well, estimated an average consumption of 9.9%E from FS, for Canadian adults >19 years [23]. Veugelers et al., 2020 also found similar estimates (13.3%E) of FS for Canadians using 2015 CCHS Nutrition data [22].

In general, the approaches used to estimate FS intake in all these studies are similar but appear to have a few differences [14,15,22,23]. One of the factors influencing the estimates is what researchers included in their definition of FS. As well, since an ingredient list is not available in the CNF, professional judgement was required when going through the different steps to estimate FS. We also used the University of Toronto’s FLIP database that has nutritional information of food and drink products in the Canadian food supply, rather than databases from other countries which other Canadian studies have used [9,14,22,23]. These methodological nuances could lead to different decisions when it comes to identifying food sources of FS and, thus, the impact population-level estimates. We also observed differences in statistical methodologies that could lead to slightly different estimates from ours. For example, in our study we used the univariate approach from the NCI methodology to estimate %E from FS (i.e., usual intake of ratios), while other researchers used the bivariate approach (i.e., ratio of usual intakes). In some cases, these two approaches could yield to different results. Additionally, our study applied certain exclusion criteria to the population, as noted in the methodology section, which differed from the exclusion criteria used by others. While there are differences in the definitions used for FS and variations in the methodologies used to estimate FS intake, these studies show similar results. The estimates based on our methodology and those of others demonstrate that, overall, Canadians consume FS above the WHO recommendation of <10%E.

Other interesting data that are useful to guide population health interventions are analyses from various age–sex and demographic groups. Overall, we found that the top for four food sources of free sugars are similar between the various age–sex groups except for adult men (≥19 years), who reported consuming more FS from sugary drinks compared to adult women.

Our results also showed that there were no important differences in the intake of FS (from both %E and g) between different BMI groups in Canadian population ≥1 year (<1% difference in %E). While these results may be unexpected, as one would expect that a high FS intake may lead to high BMI, they may be explained by under-reporting in higher BMI groups [24]. Furthermore, we found that there was no important difference between Canadians living in urban settings compared to rural settings in %E from FS (<1% difference in %E); however, the absolute amount showed a significant difference at 61.6 g vs. 54.5 g (p < 0.001). This was interesting given that Canadian living in urban centres reported consuming a mean caloric intake of 1836 calories (95% CI: 1809.1–1863.4) compared to rural respondents consuming a higher mean intake of 1970 calories (95% CI: 1913.2–2027.5). One potential factor explaining the higher absolute FS estimate in urban populations may be the greater availability of foods containing FS in urban settings, which may contribute to higher intakes of FS in urban populations compared to rural settings [25,26,27]. Our study is the first to estimate intakes of FS of various sociodemographic groups in Canada.

Canadians’ FS intakes are similar to international estimates. A national population-level health survey from Australia found that Australians consumed about 11%E from FS (about 60 g) [28]. The Australian data showed that about 52% of total sugars intake came from FS [17]. Similar to Canada, a New Zealand population-level survey showed that men consumed more FS than women [29]. Population-level surveys from the United Kingdom found British children consumed between 12–15%E from FS compared with 14–16%E for Canadian children [16,30]. These comparisons show much work remains to be done globally to meet the WHO FS recommendation in order to help reduce the risk of non-communicable diseases.

The 2019 Canada’s Food Guide (CFG) recommends limiting the consumption of highly processed products which are high in FS, sodium and saturated fats. Most foods in the top 10 sources of FS in the diet of Canadians aged ≥1 year are highly processed foods that do not meet CFG recommendations. Encouraging the consumption of foods recommended as part of the CFG and limiting the foods high in FS, sodium and saturated fats can contribute to improved dietary intakes of Canadians, while meeting the WHO’s FS recommendations.

### Limitations of the Study

There were a few limitations in this study. The use of the 2015 CNF to assess the FS intake of Canadians is challenging, since the composite database does not contain ingredient lists. Food intakes in CCHS were self-reported and, therefore, could be prone to recall bias or potential under-reporting because of social desirability. Comparison with 2004 CCHS reported data was not possible as FS estimation was not done, thus changes in consumption between 2004 and 2015 for FS in Canadians is unknown. As well, there is inherent subjectivity in this methodology even though knowledgeable Registered Dietitians applied extensive knowledge of nutrition and food composition at each of the six steps.

## 5. Conclusions

We developed a methodology to estimate FS intakes of Canadians and applied this methodology to CCHS-Nutrition (2015). We found Canadians consume about 12%E from FS; this varied between 11%E to 16%E by various age–sex groups. Adolescents aged 9–13 years and 14–18 years appear to consume the highest amount of their energy intake from FS. Similar amounts of FS intakes were observed between men and women; however, the main sources of FS differed. Only 40% of Canadians ≥ 1 year consume <10%E from FS in line with the WHO recommendation. The age groups of most concern are adolescents, since they show the lowest proportion with intakes below the <10%E from FS recommendation (less than 14% for 9–13 years and less than 18% for 14–18 years).

The main sources of FS in the diet of Canadians were “sugary drinks”, “sugars, syrups, preserves, confectionary, desserts and “baked products”. Altogether sugary drinks accounted for most of the FS intakes (40%E) among Canadians (≥1 year).

These results reinforce the need for initiatives to help Canadians, particularly children and adolescents, to reduce their FS intake. It supports Health Canada’s promotional and educational efforts on the CFG for which children and youth are priority target audiences. Encouraging the consumption of foods recommended as part of the CFG and limiting the foods high in FS can contribute to the improved dietary intakes of Canadians while meeting the WHO FS recommendations.

## Figures and Tables

**Table 1:** Mean intake of free sugars from foods in the Canadian population ≥1 year.

		Mean Intake in %E				Mean Intake in Grams			
Variable	Category	*n*	Mean	SE	*p*-Value ≠¤	*n*	Mean	SE	*p*-Value ≠¤
Overall		19,210 *	11.8	0.1		19,218	55.7	0.7	
Sex	M	9293	11.9	0.2	0.532	9297	63.8	1.1	<0.0001
	F	9917	11.7	0.2		9921	47.6	0.8	
Age groups	1–8 years	2470	13.8	0.2	<0.0001	2471	54.2	1.3	<0.0001
	9–13 years	1956	15.2	0.3		1956	74.9	1.9	
	14–18 years	1878	14.8	0.3		1878	80.4	2.5	
	≥19 years	12,906	11.1	0.1		12,913	52.9	0.8	
Age-sex groups	M: 1–8 years	1244	14.3	0.3	<0.0001	1244	58.1	1.6	<.0001
	F: 1–8 years	1226	13.3	0.4		1227	50.4	1.9	
	M: 9–13 years	1018	14.9	0.4		1018	77.7	2.6	
	F: 9–13 years	938	15.6	0.5		938	72.0	2.6	
	M: 14–18 years	897	15.0	0.5		897	91.7	3.9	
	F: 14–18 years	981	14.6	0.5		981	68.3	2.9	
	M: ≥19 years	6134	11.1	0.2		6138	61.5	1.3	
	F: ≥19 years	6772	11.0	0.2		6775	44.2	0.9	
BMI	under/normal weight	6062	12.2	0.2	0.023	6065	58.2	1.2	0.167
	at risk of/or overweight	4138	11.3	0.2		4138	54.1	1.4	
	obese	3165	11.4	0.3		3166	57.5	1.9	
Type of smoker **	Daily smoker	2023	13.3	0.4	0.001	2025	66.4	2.8	0.003
	Occasional smoker	639	11.4	0.7		640	55.4	4.4	
	None smoker	12,852	11.1	0.1		12,856	53.6	0.8	
Urban	Yes	4577	12.3	0.3	0.017	4578	61.6	1.6	<0.0001
	No	14,633	11.6	0.1		14,640	54.5	0.8	
Education	University degree/diploma	6811	11.1	0.2	<0.001	6811	52.4	1.0	0.005
	<University degree/diploma	12,372	12.2	0.1		12,380	58.0	0.9	
Adjusted household income	Q1 (Lowest)	4997	12.3	0.3	<0.001	5000	54.7	1.4	0.577
	Q2	4852	12.1	0.2		4853	56.7	1.3	
	Q3	4676	11.9	0.3		4680	57.2	1.6	
	Q4 (Highest)	4685	10.7	0.2		4685	54.4	1.4	

≠ *p*-values based on the Wald’s F test. *p*-values are based on normally transformed values of free sugars. * Eight participants reported zero energy intake; therefore, they were excluded from this analysis, ** Participants ≥ 12 years. Source: First 24 h recall from 2015 CCHS-Nutrition data.

## Data Availability

The free sugars estimates of the foods from the Canada Nutrient File (2015) are available as supplementary Materials.

## Supplementary Materials

The following are available online at https://www.mdpi.com/article/10.3390/nu13051471/s1: Table S1: Free sugars amount for foods found in the 2015 CNF.

## Appendix A

**Table A1:** *t*-tests for differences between mean intake of %E from free sugars in Canadian population ≥ 1 years.

Comparison	Comparisons	*p* Value ¥¤
Sex	M vs. F	0.532
Age groups	1–8 years vs. 9–13 years	0.001
	1–8 years vs. 14–18 years	0.947
	1–8 years vs. 19+ years	<0.0001
	9–13 years vs. 14–18 years	0.594
	9–13 years vs. 19+ years	<0.0001
	14–18 years vs 19+ years	<0.0001
Age-sex groups	M 1–8 years vs. F 1–8 years	0.518
	M 9–13 years vs. F 9–13 years	0.944
	M 14–18 years vs. F 14–18 years	1.000
	M ≥19 years. vs. ≥19 years	1.000
BMI	under/normal weight vs. at risk of/or overweight	0.042
	under/normal weight vs. obese	0.106
	at risk of/or overweight vs. obese	1.000
Type of smoker *	daily smoker vs. occasional smoker	0.056
	daily smoker vs. none smoker	0.000
	occasional smoker vs. none smoker	1.000
Urban	Y vs. N	0.017
Education	< University degree/diploma	0.000
Adjusted household income	Q1 vs. Q2	1.000
	Q1 vs. Q3	1.000
	Q1 vs. Q4	0.002
	Q2 vs. Q3	1.000
	Q2 vs. Q4	0.002
	Q3 vs. Q4	0.016

¥ Bonferroni correction applied to adjust for multiple comparisons when applicable, *p*-values are based on normally transformed values of free sugars. * Participants ≥ 12 years.

**Table A2:** Comparison of the Mean Intakes of FS When Applying the WHO Definition vs. Our Adapted WHO Definition.

Variable	Category	Realtive Mean (%E)		Absolute Mean (g)	
		Adapted WHO Definition	WHO Definition	Adapted WHO Definition	WHO Definition
Overall		11.8	11.8	55.7	54.1
Sex	M	11.9	11.9	63.8	61.8
	F	11.7	11.7	47.6	46.2
Age groups	1–8 years	13.8	13.6	54.2	51.9
	9–13 years	15.2	15.5	74.9	73.0
	14–18 years	14.8	15.1	80.4	78.3
	≥19 years	11.1	11.1	52.9	51.3
Age-sex groups	M: 1–8 years	14.3	13.9	58.1	55.4
	F: 1–8 years	13.3	13.2	50.4	48.6
	M: 9–13 years	14.9	15.2	77.7	75.7
	F: 9–13 years	15.6	15.8	72.0	70.2
	M: 14–18 years	15.0	15.2	91.7	88.9
	F: 14–18 years	14.6	14.9	68.3	66.9
	M: 19 years+	11.1	11.2	61.5	59.6
	F: 19 years+	11.0	11.0	44.2	43.0
BMI	under/normal weight	12.2	12.2	58.2	56.5
	at risk of/or overweight	11.3	11.4	54.1	52.6
	obese	11.4	11.5	57.5	56.1
Type of smoker *	Daily smoker	13.3	13.4	66.4	64.7
	Occasional smoker	11.4	11.3	55.4	53.4
	None smoker	11.1	11.2	53.6	52.0
Urban	Y	12.3	12.3	61.6	59.5
	N	11.6	11.7	54.5	52.9
Education	University degree/diploma	11.1	11.2	52.4	50.7
	<University degree/diploma	12.2	12.2	58.0	56.3
Adjusted household income	Q1 (Lowest)	12.3	12.4	54.7	53.2
	Q2	12.1	12.1	56.7	55.2
	Q3	11.9	11.9	57.2	55.4
	Q4 (Highest)	10.7	10.7	54.4	52.5

* Participants ≥ 12 years.
